# Emerging roles of PIWI-interacting RNAs in cancer molecular diagnostics and therapeutics: a molecular biosciences perspective

**DOI:** 10.3389/fmolb.2026.1775335

**Published:** 2026-04-07

**Authors:** Qianqian Yang, Jingping Wu, Yu Su

**Affiliations:** 1 Clinical Medical College, Chengdu University of Traditional Chinese Medicine, Chengdu, Sichuan, China; 2 Department of Medical Aesthetics, Affiliated Hospital of Chengdu University of Traditional Chinese Medicine, Chengdu, Sichuan, China; 3 Hangzhou Heyunjia Hospital, Hangzhou, Zhejiang, China

**Keywords:** biomarkers, cancer, early detection, molecular biosciences, PIWI-interacting RNAs

## Abstract

Cancer claims nearly 10 million lives yearly, demanding innovative diagnostics and therapies beyond surgery and chemotherapy’s limitations, such as resistance and toxicity. Next-generation sequencing has unveiled PIWI-interacting RNAs (piRNAs), 26–31 nucleotide small non-coding RNAs, as pivotal regulators in cancer pathogenesis, offering fresh biomarkers and targets from a molecular biosciences lens. Once deemed germline-exclusive for transposon silencing via PIWI proteins (PIWIL1-4), piRNAs exhibit somatic dysregulation across malignancies, driving hallmarks like proliferation, metastasis, and chemoresistance. In colorectal cancer, piR-823 fosters invasion by stabilizing HIF-1α and G6PD, correlating with poor prognosis. Gastric cancers overexpress piR-651, promoting G2/M arrest evasion; lung cancers feature PMLCPIR enhancing ITGB1-PI3K-AKT signaling; multiple myeloma leverages piR-823 for proliferation; and hepatocellular carcinoma shows PIWIL1 upregulation tied to stemness. PiRNAs’ tissue-specific signatures enable liquid biopsy detection, with panels predicting recurrence-free survival (e.g., piR-54265/STAT3 axis in CRC). Therapeutically, piRNA mimics/inhibitors (e.g., LNA-antisense against piR-L-138 in lung squamous cell carcinoma) sensitize tumors to cisplatin, while PIWI knockdown curbs metastasis preclinically. This review dissects piRNA biogenesis, oncogenic/suppressive duality, and translational promise. By bridging molecular mechanisms to clinical utility, encompassing diagnostics via plasma profiling and therapeutics like nanoparticle-delivered piRNA therapeutics, piRNAs herald a paradigm shift in precision oncology.

## Introduction

Cancer continues to pose a major global health burden, with lung cancer as the leading cause of cancer-related mortality, highlighting the critical need for advanced diagnostic and therapeutic strategies. Recent studies have increasingly highlighted the emerging roles of PIWI-interacting RNAs (piRNAs), small non-coding RNAs (24–31 nucleotides) that bind PIWI proteins, in cancer progression, including non-small cell lung cancer (NSCLC), pancreatic ductal adenocarcinoma (PDAC), and biliary tract cancers (Song et al., 2025; Liu et al., 2025; Kasik et al., 2025). PiRNAs are transcribed from piRNA clusters by RNA polymerase II and mature via Dicer-independent pathways, including primary processing and the conserved “ping-pong” amplification cycle involving PIWI clade Argonaute proteins (e.g., PIWIL1-4 in humans) (Liu et al., 2024). These piRNA-PIWI complexes silence transposons, induce epigenetic modifications like DNA methylation and histone changes (e.g., H3K9me2/3), and regulate post-transcriptional gene silencing or protein interactions to maintain genomic stability (Song et al., 2025; Garcia-Borja et al., 2024).

Once thought germline-specific, piRNAs and PIWI proteins are aberrantly expressed in somatic tumors, driving hallmarks like proliferation, invasion, metastasis, chemoresistance, and stemness, often via pathways such as AKT/mTOR, Wnt, or JAK/STAT. Examples include upregulated piR-651 and piR-823 promoting proliferation and poor survival in NSCLC and colorectal cancer, while downregulated piR-55490 or piR-211106 suppresses tumor growth; such patterns also appear in PDAC and biliary cancers (Song et al., 2025; Garcia-Borja et al., 2024; Ahmadi et al., 2024; Taghizadeh et al., 2024). Circulating and exosomal piRNAs (e.g., piR-164586, piR-26925) show high sensitivity/specificity for early NSCLC detection via liquid biopsy, with overexpression of piR-823 linked to worse overall survival (HR 3.82). PIWI proteins like PIWIL1 correlate with recurrence-free and overall survival, positioning the piRNA-PIWI axis as robust biomarkers superior to some traditional ncRNAs due to stability (Song et al., 2025; Kasik et al., 2025; Garcia-Borja et al., 2024; Taghizadeh et al., 2024).

Targeting the piRNA-PIWI axis, via inhibitors of oncogenic piRNAs (e.g., piR-651) or mimics of suppressors, enhances chemosensitivity (e.g., to cisplatin) and overcomes resistance in NSCLC models, with potential for combination therapies in precision oncology (Garcia-Borja et al., 2024; Yao et al., 2022). This review examines piRNA biogenesis, cancer-specific mechanisms, dysregulation across malignancies, and translational prospects as biomarkers and targets from a molecular biosciences viewpoint.

## Biogenesis and biological functionality

### piRNA biogenesis and its regulatory mechanisms

Non-coding RNAs (ncRNAs), including microRNAs (miRNAs) (Chaleshtori et al., 2025), long non-coding RNAs (lncRNAs) (Mattick et al., 2023), circular RNAs (circRNAs) (Dorostgou et al., 2022), and PIWI-interacting RNAs (piRNAs) (Wang et al., 2023), have emerged as key regulators of gene expression in cancer and development. Contemporary research on piRNAs concentrates on organisms such as *Drosophila melanogaster*, *Mus musculus*, and *Caenorhabditis elegans*. PiRNAs modulate gene expression via interactions with PIWI, encompassing posttranscriptional gene silencing (PTGS), multiprotein interactions, and transcriptional gene silencing (TGS) (McEnany et al., 2022). These complexes facilitate TGS via histone-modifying enzymes and PTGS through mRNA cleavage (De Fazio et al., 2011; Reuter et al., 2011; Wang et al., 2015).

### Generation of precursor piRNA from piRNA cluster

PiRNAs are classified into three categories: transposon-derived, mRNA-derived, and lncRNA-derived. They are derived from uni-strand clusters in the nucleus of *D. melanogaster*, and both male and female germline cells contain dual-strand clusters. Parenthetically, uni-strand clusters are typically expressed in somatic follicle cells, whereas dual-strand clusters are the germline standard. This distinction is vital for understanding the different regulatory requirements of the two cell types. In order to prevent premature termination and facilitate their cytoplasmic translocation through the nuclear RNA export factor or DEAD-box helicase U2AF65-associated protein, the Rhino-Deadlock-Cutoff (RDC) complex helps with the transcription of precursor piRNAs (Ariura et al., 2024).

### Formation of mature piRNA/PIWI complexes through two distinct pathways

The principal pathway of *D. melanogaster* entails the translocation of precursor piRNAs to Nuage, where they undergo cleavage by Zucchini endonuclease and Minotaur, which Gasper plays a pivotal role in this step. This process produces mature piRNAs featuring uridine at the 5′-terminus. Hua Enhancer 1 (Hen1) methylates the 2′-hydroxy group at the 3′-terminus of piRNA, yielding a mature piRNA/PIWI complex. This modification is essential for piRNA stability, distinguishing them from miRNAs which typically lack this mark in animals (Izumi et al., 2020). Aub engages with antisense piRNAs in the secondary pathway, cleaves transposon elements, and produces secondary sense piRNAs that associate with Ago3 complexes. The reciprocal cleavage mechanism generates an amplification loop that enhances piRNA levels, referred to as the ping-pong pathway (Santos et al., 2023).

### Transcriptional gene silencing guided by the piRNA/PIWI complex

PiRNA/PIWI complexes, in conjunction with Asterix (Gtsf1) and Panoramix, commence TGS through their interaction with target precursor mRNA. Eggless and Windei facilitate repressive di- or tri-methylation of histone 3 lysine 9. Panoramix acts as the ‘molecular bridge’ between the piRNA/PIWI complex and the epigenetic machinery (Eggless/Windei). Lsd1 removes H3K4me2 modifications, whereas Heterochromatin protein 1 (HP1) creates heterochromatin. Gene methylation through the piRNA/PIWI complex, which recruits DNA methyl transferases, is a component of epigenetic regulation. It is worth considering that *Drosophila* does not utilize DNA methylation for this purpose. In prostate cancer, PiR-31470/PIWIL4 enhances GSTP1 silencing (Czech et al., 2018; Jia et al., 2022; Ma et al., 2024).

### piRNA/PIWI complex-guided posttranscriptional gene silencing

Ping-pong amplification is essential in PTGS, as it generates silencing triggers against active elements and can directly cleave transposon RNA. PiRNA and PIWI form the piRNA-induced silencing complex (piRISC), which finalizes PTGS through mRNA cleavage or deadenylation; this is achieved through the recruitment of the CCR4-NOT complex. In both *Drosophila* and mammals, the interaction between PIWI proteins and the CCR4-NOT scaffold is the definitive step that triggers mRNA instability and subsequent degradation, distinguishing this pathway from direct ‘Slicer’ cleavage. PiRISC engages the CCR4-NOT complex in *D. melanogaster* and CAF1 in *M. musculus* (Czech and Hannon, 2016; Loubalova et al., 2023; Claro-Linares and Rojas-Rios, 2025; Rojas-Rios et al., 2024). It also cleaves and degrades mTOR mRNA in lung adenocarcinoma and CCL3 expression in clear cell renal carcinoma (Fan et al., 2020; Quinn et al., 2024).

### Multiprotein interactions guided by the piRNA/PIWI complex

The piRNA/PIWI complex constitutes a tertiary regulatory mechanism in cancer therapy. The compound engages with HSF1, facilitating Ser326 phosphorylation, which augments cell proliferation and inhibits apoptosis, and interacts with STAT3, promoting its phosphorylation and imparting cancer resistance in CRC cells (Jia et al., 2022; Liu et al., 2019).

### piRNA in cancer development

Dysregulated piRNAs are associated with the onset of numerous diseases, including cancer, hypertension, and myocardial infarction. Cancer progression and development encompasses four fundamental biological functions: proliferation, metastasis, cell cycle arrest, and genetic or epigenetic variation. Notwithstanding various anti-cancer treatments, the advancement of cancer continues to be a substantial concern. Research demonstrates that different piRNAs contribute to cancer progression, with additional studies uncovering both oncogenic and tumor-suppressive functions in these mechanisms (Lopez-Jimenez and Andres-Leon, 2021; Turko et al., 2025) (Tables 1, 2). Here, we explore the role of piRNAs, as well as exosomal piRNAs, in diverse human cancer systems, their association with cancer hallmarks, and their potential as diagnostic tools, therapeutic targets, and prognostic biomarkers.

**TABLE 1:** Molecular role of piRNAs in various cancers.

Cancer type	piRNA	Molecular mechanism	Expression in cancers	References
Lung cancer	piR-651	Enhance cellular proliferation and correlate with TNM stages	Downregulated	Yao et al. (2016)
Gastric cancer	piR-FR387750	Correlated with recurrence-free survival	Upregulated	Martinez et al. (2016)
Colorectal cancer	piR-1245	Facilitated cellular proliferation, enhanced migration and invasion, and inhibited apoptosis by binding to its downstream targeted mRNA in nuclear exosomes, correlated with poor differentiation, TNM classification, and diminished overall survival	Upregulated	Weng et al. (2018)
Breast cancer	piR-DQ598677	Utilize pi-RISC to degrade specific genes such as miRNAs	Downregulated	Hashim et al. (2014)
Breast cancer	piR-021285	ARHGAP11A methylation suppressed cell proliferation and invasion	Downregulated	Fu et al. (2015)
Breast cancer	piR-932	Prompted the epithelial-mesenchymal transition by promoting the methylation of the CpG island in the promoter region of Latexin	Upregulated	Zhang et al. (2013)
lung cancer	piR-55490	Suppressed LC cell and cancer growth by binding to the 3′ UTR of mTOR mRNA	Downregulated	Peng et al. (2016)
Colorectal cancer	piR-015551	The onset of colorectal cancer was affected by a genetic mutation	Upregulated	Chu et al. (2015a)
Gastric cancer	piR-FR064000	Positively correlated with overall survival	Upregulated	Martinez et al. (2016)
Gastric cancer	piR-823	Inhibited tumor cell proliferation and prompted a “stem-like” phenotype in cells by diminishing the methylation of oncogenic genes	Downregulated	Liu et al. (2006)
Gastric cancer	piR- FR222326	Positively correlated with overall survival	Upregulated	Martinez et al. (2016)
Colorectal cancer	piR-54265	The modulation of STAT3 phosphorylation can enhance proliferation, and metastasis, and diminish progression-free survival, consequently leading to resistance against anti-tumor therapies	Upregulated	Mai et al. (2018)
Kidney cancer	piR-57125	Effectively curtailed the metastatic advancement of cancer	Downregulated	Busch et al. (2015)
Gastric cancer	piR- FR222326	Exhibits a positive correlation to the overall survival rate	Upregulated	Martinez et al. (2016)
Breast cancer	piR-021285	By inducing ARHGAP11A methylation to impede cell proliferation and invasion	Downregulated	Fu et al. (2015)
Colorectal cancer	piR-823	Facilitated cell proliferation and suppressed apoptosis by augmenting HSF1 phosphorylation at Ser326 and inducing Stat3 phosphorylation	Upregulated	Yin et al. (2017)
Glioblastoma	piR-DQ593109	Interacted with MEG3 to enhance the permeability of the blood-tumor barrier, thereby promoting the delivery of therapeutics into the glioma microenvironment	Downregulated	Shen et al. (2018)
Kidney cancer	piR-30924, piR-38756	Correlated to the cancer metastasis	Upregulated Downregulated	Li et al. (2015)
Ovarian cancer	piR-52207	By binding of specific mRNA (NUDT4, MTR, EIF2S3, MPHOSPH8) facilitated cell proliferation, migration, and tumorigenesis	Upregulated	Singh et al. (2018)
Fibrosarcoma	piR-39980	By interacting with RRM2 to impede cell proliferation	Downregulated	Müller et al. (2015)
Kidney cancer	piR-32051, piR-39894, piR-43607	Involved in the progression of advanced renal cell carcinoma, its metastasis, and the associated survival rates	Upregulated	Li et al. (2015)
Glioblastoma	piR-651	Correlated with reduced disease-free and overall survival in patients with classical Hodgkin lymphoma	Upregulated	Cordeiro et al. (2016)
Lung cancer	piR-34871, piR-52200, piR-46545, piR-35127	RASSF1C expression was associated with enhanced cell proliferation and colony formation through the reduction of AMPK phosphorylation within the ATM-AMPK-p53-p21cip pathway	Upregulated Downregulated	Reeves et al. (2017)
Hepatocellular carcinoma	Hsa-piR-013306	Engaged in the hepatic carcinogenesis process	Upregulated	Rizzo et al. (2016)
Glioblastoma	piR-30188	Suppressed tumor cell growth, motility, and infiltration, while facilitating apoptosis through its interaction with OIP5-AS1	Downregulated	Liu et al. (2018a)
Pancreatic cancer	piR-017061	Not determined	Downregulated	Müller et al. (2015)
Hepatocellular carcinoma	piR-Hep1	The activation of the PI3K/AKT signaling pathway can enhance cell proliferation and invasion	Upregulated	Law et al. (2013)
Glioblastoma	piR-8041	Interacted with MEG3 enhanced the permeability of the blood-tumor barrier, thereby promoting the delivery of therapeutics into the glioma microenvironment	Downregulated	Jacobs et al. (2018)
Hepatocellular carcinoma	piR-LLi-24894	Linked to primary stages of hepatocellular carcinoma	Upregulated	Rizzo et al. (2016)
Glioblastoma	piR-30188	Suppressed tumor cell proliferation, invasion, and migration, and facilitated apoptosis by binding to OIP5-AS1	Downregulated	Liu et al. (2018a)

**TABLE 2:** The molecular role of PIWI proteins in various cancer types.

Cancer type	PIWI	Expression in cancers	Molecular mechanism	References
Cervical cancer	PIWIL2	Upregulated	It prompted H3K9 acetylation while diminishing H3K9 trimethylation	Wang et al. (2016)
Renal cell cancer	PIWIL1	Downregulated	These biomarkers may function as prospective prognostic indicators in patients with RCC	Iliev et al. (2016a)
Renal cell cancer	PIWIL2	Downregulated	Associated with poor survival	Iliev et al. (2016a)
Glioblastoma	PIWIL2	Upregulated	Associated with poor survival	Li et al. (2017)
Gastric cancer	PIWIL1	Upregulated	Modulate the signaling pathway associated with gastric cancer	Araújo et al. (2018)
Gastric cancer	PIWIL3	Upregulated	The regulation of the JAK2/STAT3 signaling pathway is essential for numerous biological processes	Yin et al. (2017)
Non-small cell lung cancer	PIWIL2	Upregulated	The expression levels of CDK2 and Cyclin A have risen	Qu et al. (2015)
Endometrial cancer	PIWIL1	Upregulated	DNA methylation is a significant target for the development of a new therapeutic approach	Chen et al. (2015)
Triple-negative breast cancer	PIWIL4	Upregulated	The activation of TGF-β, MAPK/ERK, and FGF signaling, consequently inhibiting immune recognition	Wang et al. (2016)
Colorectal cancer	PIWIL1	Upregulated	The utilization of a molecular marker is essential for forecasting the prognosis of CRC patients	Sun et al. (2017)
Multiple myeloma	PIWIL3	Upregulated	The proliferation and metastatic characteristics of multiple myeloma	Gambichler et al. (2017)
Lung cancer	PIWIL1	Upregulated	DNA hypomethylation	Xie et al. (2018)
Glioblastoma	PIWIL3	Downregulated	The regulation of the PIWIL3/piR-30,188/OIP5-AS1/miR-367-3p/CEBPA/TRAF4 pathway is essential for numerous biological processes	Liu et al. (2018a)
Colorectal cancer	PIWIL1	Upregulated	As a molecular marker is essential for forecasting the prognosis of CRC patients	Sun et al. (2017)

### piRNAs and cellular proliferation, invasion, and motility

Cancer represents a significant characteristic of unregulated cell proliferation, with piRNAs serving an essential function in this mechanism. Through the expression of cyclin D1 and CDK4, piR-651 promotes cell proliferation and tumorigenesis in lung cancer tissues and cell lines, according to deep sequencing analysis. PiR-8041 is downregulated in glioblastoma multiforme, leading to increased cell proliferation and tumor growth inhibition in a dose-dependent manner (Yao et al., 2022). PiR-30924 and piR-38756 exhibit elevated expression in metastatic tumors, whereas piR-57125 demonstrates reduced expression in these tumors. The PIWI subfamily, comprising piRNA-binding proteins, also plays a role in the metastatic characteristics of cancer. The downregulation of PIWIL1 markedly diminishes the growth, infiltration, and motility of hepatocellular carcinoma (HCC) (Zhang et al., 2022). PIWIL1 is a prevalent gene in endometrial cancer tissues, playing a crucial role in preserving stem cell-like properties. The identification of PIWI proteins associated with EMT markers (e.g., Snail, Slug, or Zeb1) may enhance therapeutic approaches and modulate tumor metastasis during the pivotal stage of tumorigenesis. Investigations into piRNAs have demonstrated their capacity to suppress immune regulation, including IL expression and the development of Th2 T-lymphocytes. One study indicated that piR-30840 enhances the stemness of multiple myeloma stem cells, while others advocate for further investigation of piRNAs within the tumor microenvironment (Limanowka et al., 2024).

### The functional role of piRNAs in the cell cycle

Cell cycle arrest, triggered by aberrant regulatory factors, is a pivotal element of cancer development, with PiRNAs and PIWI proteins significantly contributing to this phenomenon. In colorectal cancer cells, the antisense sequence suppressed piR-823, leading to cell cycle arrest and an increased proportion of cells in the G1 phase. PIWIL1 was found to be excessively expressed in gastric tissues and cell lines, while its downregulation significantly inhibited cell growth and initiated cell cycle arrest during the G2/M phase. CyclinD1, a crucial regulator of cell cycle advancement, governs the secretion of piRNAs in breast cancer (Yin et al., 2017).

### The potential role of piRNAs in cancer epigenetic

Epigenetic modifications encompass changes in DNA methylation, histone modifications, and chromatin remodeling. DNA methylation, particularly CpG island methylation, has been associated with cancer, potentially leading to the inactivation of oncogenes. PiRNAs are linked to CpG island methylation, as demonstrated in breast cancer, where the pro-apoptotic regulator ARHGAP11A was inhibited by PiR-021285. By facilitating the methylation of CpG islands in the Latexin promoter region, PiR-932 and PIWIL2 formed a complex that reduces the expression of Latexin. In order for cervical epithelial cells to develop into tumor-initiating cells, PIWIL2 is necessary. DNA methylation is primarily facilitated by DNA methyl transferases, which are categorized into two principal classes: DNMT3 and DNMT1. PiRNAs and PIWI proteins are intricately associated with histone modifications, as PIWI proteins directly affect histone modifications at piRNA targets. Ubiquitination is essential for protein localization, metabolism, regulation, and degradation. PiRNAs in cancer frequently engage with various cancer hallmarks, including the inhibition of tumorigenesis, the promotion of cell cycle regulators, apoptosis-associated proteins, and the secretion of vascular endothelial growth factor. These functions may entail intricate mechanisms that necessitate additional investigation (Jia et al., 2022; Assumpcao et al., 2015; Liu J. et al., 2018).

### The regulatory function of piRNAs in chemotherapy treatment

Chemotherapy is the predominant treatment for malignant tumors (Canowitz et al., 2026), and certain piRNAs have been identified to augment chemoresistance or sensitivity. Research has identified piR-L-138 as a critical factor in cisplatin resistance among patients with lung squamous cell carcinoma, while piR-36712 in breast cancer demonstrated synergistic anticancer effects when combined with chemotherapy agents. Overexpression of piR-54265 in advanced colorectal cancer cells exhibited resistance, whereas knockdown of piR-54265 demonstrated sensitivity to 5-FU and oxaliplatin. The revelation that piRNAs can modulate chemosensitivity presents promising prospects for cancer treatment; however, additional investigation is required to elucidate the underlying mechanisms (Wang et al., 2017; Dasari and Tchounwou, 2014).

### The regulatory function of piRNAs in different types of cancer

Recent studies indicate a significant correlation between piRNAs, tumor promoters or suppressors, and tumor cell malignancy and clinical stage across multiple cancers, elucidating their mechanisms.

### Breast cancer (BC)

**FIGURE 1:**
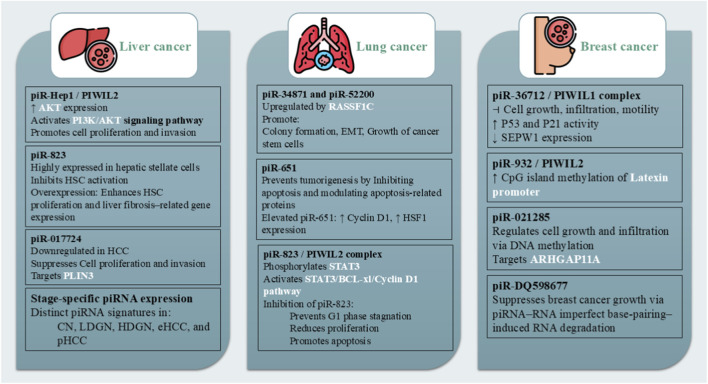
This figure summarizes the regulatory functions of piRNAs in breast, lung, and liver cancers. In breast cancer, piRNAs regulate proliferation, invasion, cell cycle arrest, and epigenetic modification. In lung cancer, piRNAs modulate apoptosis, EMT, and oncogenic signaling pathways. In liver cancer, piRNAs influence PI3K/AKT signaling, disease progression, fibrosis, and exhibit diagnostic potential through stage-specific and exosomal expression patterns.

Breast cancer is the most prevalent cancer diagnosed worldwide and the primary cause of cancer-related deaths among women. Tumor size and metastasis are inversely correlated with the piR-36712/PIWIL1 complex, which inhibits cell growth, infiltration, and motility. Increased expression of PiR-36712 exhibits synergistic anticancer effects with doxorubicin and paclitaxel, reducing SEPW1 expression, increasing P53 and P21 activities, and inducing G1 cell cycle arrest. While piR-932 and PIWIL2 increase the methylation of the latexin promoter CpG island in breast cancer stem cells, piR-021285 regulates cell growth and infiltration through DNA methylation. Through piRNA-RNA imperfect base-pairing-induced RNA degradation, PiR-DQ598677 suppresses the growth of breast cancer, underscoring the importance of focusing on tumor-suppressive non-coding RNAs as potential therapeutic targets (Tan et al., 2019; Jeong et al., 2021) (Figure 1).

### Lung cancer

Lung cancer is a common cancer that is one of the neoplasms with lowest 5-year survival rate and the highest incidence and mortality rates of any cancer. The expression of PiR-34871 and piR-52200 is upregulated by the tumor promoter RASSF1C, which promotes colony formation, EMT, and the growth of liver cancer stem cells. By inhibiting apoptosis and changing the expression of proteins linked to apoptosis, PiR-651 prevents tumorigenesis in human lung cancer cells, while it also acts as an oncogene in NSCLC. Treatment slows the growth of tumors by increasing the expression of proteins linked to apoptosis. The expression of oncogenes like cyclin D1 and HSF1, a transcription factor that controls heat shock proteins, may be enhanced by elevated levels of PiR-651 in NSCLC. The STAT3/BCL-xl/cyclinD1 signaling pathway may be activated by the PiR-823/piwil2 complex phosphorylating STAT3, which could start the development of colorectal cancer. Treatment with a PiR-823 inhibitor prevents G1 phase stagnation, reduces cell division, and promotes apoptosis (Liu et al., 2019) (Figure 1).

### Liver cancer

Liver cancer ranks as the second foremost cause of cancer-related mortality in men across both developing and developed countries. The research indicates that increased piRHep1 in liver cancer may promote proliferation and invasion through its interaction with PIWIL2, leading to the upregulation of AKT in the PI3K/AKT signaling pathway (Liu et al., 2019; Ameli et al., 2022). In 2016, Rizzo et al. examined the piRNA expression profile of various stages of hepatocellular carcinoma (HCC) using next-generation sequencing. They discovered a distinct piRNA expression signature for early hepatocellular carcinoma (eHCC) and progressed hepatocellular carcinoma (pHCC), as well as a distinct signature for low-grade dysplastic nodule (LDGN) and high-grade dysplastic nodule (HDGN), in resection samples taken from 17 patients with multiple nodules (Tierno et al., 2024). Koduru et al. (2018) discovered in 2018 that each pathological stage linked to HCC has a distinct piRNA expression profile, suggesting that piRNoma undergoes dynamic changes as the disease advances. The study found particular dysregulated piRNAs in combined hepatocellular carcinoma (CN), LDGN, HGDGN, eHCC, and pHCC samples using RNA-seq datasets from the NIH Short Read Archive, making their application as clinical tools more challenging. Tang et al. (2018) investigated the role of piRNAs in liver diseases that cause mice to develop HCC. They discovered that primary hepatic stellate cells (HSCs) had high expression of piR-823, which prevented HSC activation. PiR-823 may be an early therapeutic target for preventing HCC, as overexpression enhanced HSC proliferation and the synthesis of genes for the advancement of liver fibrosis. According to Wu et al. (2023), piR-017724 is markedly downregulated in 45 HCC tissues and is associated with an advanced tumor stage and a poor prognosis. In HCC cell lines, its oncosuppressive function was verified; it prevented invasiveness and cell proliferation but not apoptosis. According to functional analyses, piR-017724 may suppress PLIN3, a crucial regulator of the liver’s signaling, membrane trafficking, protein degradation, and gene expression. In 2023, Rui et al. (2023) evaluated the diagnostic potential of serum exosome piRNA levels in 125 HCC patients and 44 healthy controls. They confirmed the diagnostic potential of five upregulated piRNAs in two cohorts and discovered 253 dysregulated piRNAs in tumor exosomes (Figure 1).

### Hematological malignancies

Myeloma, a hematological malignancy marked by the proliferation of malignant plasma cells in the bone marrow, is associated with the increased expression of PiRNA-823 in both patients and cell lines. Inhibition of PiRNA-823 diminishes DNA methylation, reactivates p16INK4A, and affects the proliferation, apoptosis, and cell cycle dynamics of multiple myeloma cells. G-MDSCs augment the survival and stemness of MM cells. PiRNA-823 is crucial for the reprogramming of endothelial cells within tumors, increasing their responsiveness to growth factors and their resistance to apoptotic signals, thus facilitating the proliferation of multiple myeloma cells. PiR-651, a gene located in the lymph nodes of patients with classical Hodgkin lymphoma, is essential for forecasting clinical outcomes and differentiating between responders and non-responders to initial therapy (Yan et al., 2015).

### Glioblastoma

**FIGURE 2:**
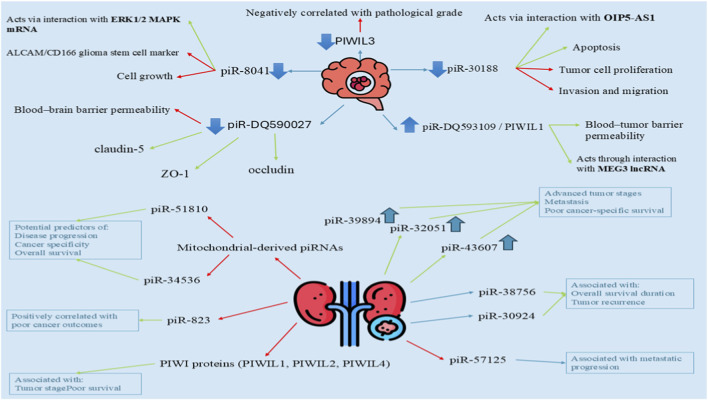
Regulatory roles of piRNAs in glioblastoma and kidney cancer. This schematic summarizes the dysregulated piRNAs and PIWI proteins involved in glioblastoma and renal cell carcinoma (RCC), highlighting their molecular interactions and associated biological effects. In glioblastoma (upper panel), downregulated piRNAs such as piR-8041 and piR-30188 suppress tumor cell growth, invasion, and migration, while promoting apoptosis through interactions with ERK1/2 MAPK mRNA and OIP5-AS1, respectively. PIWIL3 expression is negatively correlated with pathological grade. PiR-DQ593109/PIWIL1 and piR-DQ590027 modulate blood–tumor or blood–brain barrier permeability via interactions with MEG3 lncRNA and tight junction proteins (ZO-1, occludin, claudin-5). In kidney cancer (lower panel), dysregulated piRNAs are associated with tumor progression and clinical outcomes. PiR-39894, piR-32051, and piR-43607 are linked to advanced tumor stages, metastasis, and poor cancer-specific survival, while piR-30924 and piR-38756 correlate with overall survival duration and tumor recurrence. PiR-57125 is associated with metastatic progression. Mitochondrial-derived piRNAs (piR-34536 and piR-51810) are potential predictors of disease progression, cancer specificity, and overall survival. Elevated urinary piR-823 and downregulation of PIWI proteins (PIWIL1, PIWIL2, PIWIL4) are associated with poor outcomes and tumor stage in RCC. Arrows indicate promotion or upregulation, whereas blunt or opposing arrows indicate inhibition or downregulation, as described in the text.

Glioblastoma, a malignant and invasive intracranial neoplasm, is the most aggressive and infiltrative brain tumor. Expression levels of PiR-30188 and PIWIL3 are diminished and exhibit a negative correlation with the pathological grade of glioma. PiR-30188 inhibits tumor cell proliferation, invasion, and migration while facilitating apoptosis through its interaction with OIP5-AS1. PiR-8041 is downregulated in glioblastoma multiforme and diminishes cell growth by interacting with the mRNA of ERK1/2 mitogen-activated protein kinase (MAPK). The PiR-8041 treatment reduces the expression of the ALCAM/CD166 glioma stem cell marker and suppresses the proliferation of the A172 glioma cell line. PiRNADQ593109/PIWIL1 in glioma endothelial cells enhances blood-tumor barrier permeability through interaction with MEG3 lncRNA, facilitating the delivery of therapeutic agents into the glioma microenvironment. PiR-DQ590027 represents a compelling therapeutic target for glioma, as it is minimally expressed in glioma-conditioned endothelial cells; however, its overexpression may reduce ZO-1, occludin, and claudin-5 levels, thereby enhancing the permeability of the normal blood-brain barrier conditioned by glioma (Jacobs et al., 2018; Liu et al., 2021) (Figure 2).

### Osteosarcoma

The incidence of osteosarcoma, a common primary bone tumor, is comparatively stable in individuals aged 0–29. The postoperative survival rate is only 15%–25%, and the rates of disability and metastases are high, along with a poor functional recovery. 80%–90% of patients pass away as a result of distant metastases. Although its etiology and pathogenesis mechanisms are unclear, recent research indicates that piRNAs are important in the development of osteosarcoma. By targeting serpin family B member 1 (SERPINB1) and activating matrix metalloproteinase-2, piR-39980 overexpression in human osteosarcoma cell lines (143B and HOS) enhanced cell invasion, migration, and proliferation. By upregulating SERPINB1, inhibiting piRNA-39980 led to chromatin condensation and cell death restoration, indicating piR-39980 as a potential therapeutic target and prognostic indicator (Vijayamurugan and Bakhshi, 2014).

### Pancreatic duct cancer (PDAC)

By co-activating the APC/C E3 complex, which targets pinin and increases the potential for PDAC to spread, PIWIL1 causes anaphase in human PDAC. This differs from the elimination of PIWIL1 during spermatogenesis, indicating that PIWILs may also have an oncogenic role in PDAC and act as co-activators in cancerous cells (Li et al., 2020).

### Bladder cancer

In bladder cancer, piRABC is downregulated and DQ585569 is significantly upregulated. By targeting the 3′-UTR of tumor necrosis factor mRNA, PiRABC can inhibit the development of bladder cancer. PiRABC also controls cell proliferation, colony formation, and apoptosis (Chu et al., 2015b).

### Endometrial carcinoma

Endometrial carcinoma is found to have elevated levels of recently discovered piRNAs, hsa-piR-020829, hsa-piR-019914, and hsa-piR-016735, which may be new biomarkers for the disease’s early detection (Ravo et al., 2015).

### Renal cell carcinoma (RCC)

Renal cell carcinoma (RCC) presents significant challenges in detection and treatment, constituting 2.4% of all adult malignancies globally. Because it is asymptomatic, early detection is difficult, and diagnosis and treatment are still difficult. With 70%–80% of cases, renal clear cell carcinoma (ccRCC) is the most prevalent subtype of RCC. The piRNA cluster on chromosome 17 produces PiR-43607, PiR-32051, and piR-39894, which are upregulated in advanced tumor stages, metastasis, and cancer-specific survival, whereas their expression diminishes in renal cell carcinoma tissue. PiRNAs may function as biomarkers for the diagnosis, treatment, and prognosis of RCC; however, additional research is required to elucidate their mechanisms (Ameli et al., 2022).

Prognostic biomarkers may be produced by aberrant expression of piRNAs, including piR-30924, piR-57125, and piR-38756, in primary and metastatic ccRCC tissues. These piRNAs have been linked to overall survival duration and tumor recurrence, which may enhance prognostic data for ccRCC patients. Because of their downregulated expression in ccRCC tissues but not in serum, two mitochondrial-derived piRNAs, piR-34536 and piR-51810, may be used as predictive markers to identify the progression of ccRCC, the specificity of the cancer, and the overall survival duration. A strong correlation exists between ccRCC metastasis and abnormal expression levels of piR-32051, piR-39894, and piR-43607. In patients with RCC, urinary piR-823 expression is elevated and positively correlated with poor cancer outcomes. In RCC, PIWIL1, PIWIL2, and PIWIL4 are downregulated and associated with poor survival and tumor stage. Because of their intricate mechanisms, these biomarkers may be helpful prognostic biomarkers, but more research is required. The pathophysiology of RCC is significantly influenced by PIWI-like proteins; the expression of PIWI-like 1 is linked to distant metastases and tumor staging, while positivity indicates a shorter cancer-specific survival (Busch et al., 2015; Iliev et al., 2016a; Zhao et al., 2019; Li et al., 2015; Stöhr et al., 2019) (Figure 2).

### Prostate cancer

PiRNAs are important in many types of cancer, including prostate cancer. Biochemical recurrence (BCR) in prostate cancer is linked to specific piRNAs that help identify high-risk and low-risk patients. Three piRNAs—hsa-piR-000627, hsa-piR-005553, and hsa-piR-019346—have 343 common targeting genes and are linked to BCR, suggesting that piRNAs control carcinogenic processes during the development of prostate cancer. PCDH9, a tumor suppressor that targets piRNAs piR-001773 and piR-017184, is downregulated in prostate cancer. By binding to PCDH9’s 3′-UTR sites, these piRNAs can stop tumor growth both *in vivo* and *in vitro*. Prostate tumor growth may be inhibited by therapeutically suppressing these piRNAs. The investigation into the connection between piRNA and tumor suppressors provides important new information about the mechanisms underlying prostate cancer and establishes the foundation for future prognostic and treatment approaches (Zuo et al., 2019; Zhang et al., 2020).

### Gastric cancer

More than 1,000,000 new cases and 769,000 deaths from gastric carcinoma (GC) were reported in 2020, making it the third most common cause of cancer-related deaths worldwide. Some patients receive a diagnosis in the middle or late stages of GC, although it is asymptomatic in the early stages. The degree of GC malignancy is closely correlated with piRNA expression, which is higher in gastric carcinoma tissues than in normal mucosa tissues. In GC tissues, piRNAs such as PiR-651 and piR-823 exhibit differential expression, with PiR-651 being upregulated in a variety of cell lines and cancers. Cell cycle and growth in GC cells can be inhibited by PiR-823, which is downregulated in GC tissues. Compared to healthy individuals, GC patients have lower levels of PiR-651 and piR-823 in their blood. Because of their extremely sensitive positive detection rates, they are helpful biomarkers for GC diagnosis. By manipulating these piRNAs therapeutically, GC development and occurrence may be effectively inhibited. Potential use as markers for GC metastasis monitoring is suggested by the significantly higher serum expression levels of piR-004918 and piR-019308 in metastatic GC patients compared to those without metastases (Martinez et al., 2016; Lin et al., 2019; Wang et al., 2014; Cui et al., 2011; Ge et al., 2020).

### Other types of cancer

Fibrosarcoma, a rare and aggressive soft tissue sarcoma, demonstrates genetic complexity and metastasizes swiftly. PiR-39980, a tumor suppressor, suppresses RRM2 expression by interacting with its 3′-UTR. Downregulation of RRM2 impedes cell proliferation and Bcl-2 modulation, whereas PiR-52207 and PiR-33733 are upregulated in endometrioid and serous ovarian cancer, facilitating cell proliferation and tumorigenesis. PiRNAs, such as PiR-017061, play a role in the oncogenesis of ovarian cancer and may serve as therapeutic targets in pancreatic cancer, although the underlying mechanisms remain inadequately elucidated (Das et al., 2021).

### Cancer-related piRNA deregulation or variation

The quantity of piRNAs in various species and cells has grown since the publication of piRNAQuest in 2014. A thorough catalog of human, mouse, and rat piRNAs was intended in order to comprehend their genomic location, overlaps, and relationships with other lncRNAs. Originally primarily used in germline cells for transposon silencing and maintaining gene integrity, piRNAs have since been found in somatic cells across a wide range of species. The study was extended to 25 new species using the latest version of piRNAQuest, piRNAQuest V.2, which also concentrated on novel topics like the directionality of piRNA clusters, expression in both healthy and diseased tissues, and targets among lncRNAs and protein-coding genes. Research has demonstrated that piRNA clusters are the primary source of piRNA biogenesis. A density-based clustering method is being explored in order to comprehend the distribution of piRNAs and how they form. This method aids in locating primary biogenesis hotspots. PiRNA synthesis and target silencing also depend on secondary biogenesis through ping-pong amplification. The study visualizes the ping-pong signature within piRNAs and focuses on 10-nt overlap signature of the ping-pong cycle among piRNAs. The human chromosome 15 contains the greatest number of piRNAs (Ghosh et al., 2022).

To comprehend piRNA-mediated gene regulation, the study examines piRNA expression profiles in 21 disease systems as well as data from normal tissue. PiRNAs are regulated differently in healthy and diseased states; higher piRNA expression is correlated with lower target expression. The scientists anticipate that piRNA targets will likely serve as both therapeutic targets for illnesses like cancer and biomarkers for early diagnosis. Additionally, they forecast piRNA targets in mRNAs and lncRNAs during the developmental stages of the mouse testis. The study examined the differences in piRNA expression across seven cancer systems and asthenozoospermia using DESeq. Certain piRNAs, including hsa_piRNA_9871 and hsa_piRNA_27200, were found to be upregulated in lung and breast cancer, respectively, according to the analysis. Furthermore, the study found that the upregulated piRNAs hsa_piRNA_7806 and hsa_piRNA_31147 encouraged colon and renal cancer proliferation and invasiveness. These results corroborate the general expression profile of piRNAs across a range of illnesses (Ghosh et al., 2022). A comprehensive and easy-to-use database for piRNAs, piRNAQuest V.2 offers both new and pre-existing features. To preserve the expanding piRNA knowledgebase, new features and newly annotated piRNAs will be added on a regular basis.

### piRNAs as diagnostic biomarkers in cancer

Liquid biopsy provides convenience, dependability, and enhanced patient acceptance for the advancement of cancer biomarkers. Research identifies anomalous piRNAs in the bodily fluids of cancer patients, including piR-823. The expression of PiR-651, associated with cell proliferation and cell cycle arrest, exhibited upregulation in gastric cancer tissues while showing downregulation in the peripheral blood of gastric cancer patients. PiR-1245, identified in the gastric juice of gastric cancer patients with unfavorable overall survival, possesses potential as a diagnostic and prognostic biomarker. PiR-823 is predominantly located in extracellular vesicles (EVs) derived from the peripheral blood of multiple myeloma (MM) patients and MM cells. EVs are essential for intercellular communication and the tumor microenvironment, whereas encapsulated piRNAs, which exhibit stability in bodily fluids, serve as promising biomarkers for cancer diagnosis and prognosis. PiRNAs exhibit enhanced diagnostic efficacy relative to traditional markers in differentiating malignant nodules from benign ones (Limanówka et al., 2023). Serum piR-5937 and piR-28876 markedly distinguish colon cancer from healthy controls, exhibiting alterations in 71% and 69% of patients, respectively, in contrast to 48% and 26% in other patients. The combination of CEA, CA199, and both piRNAs achieved a diagnostic sensitivity of 86%, indicating that the integration of biomarkers may improve diagnostic precision (Vychytilova-Faltejskova et al., 2018).

The early identification and intervention of cancer are essential for its prognosis. PiRNAs, a class of small non-coding RNAs, are pivotal in cancer diagnosis and therapy. MicroRNAs associated with tumors in peripheral blood have been recognized as biomarkers for cancer diagnosis. PiRNAs, akin to miRNAs, exhibit stability and resistance to degradation in bodily fluids, readily traversing cell membranes into circulation, and are associated with aggressive biological behaviors. Blood specimens are extensively utilized as a non-invasive diagnostic approach in clinical practice. The study discovered that the peripheral blood levels of PiR-651 and piR-823 in GC patients are lower than those of healthy controls. These piRNAs outperform widely used biomarkers in terms of GC screening sensitivity. According to ROC curve analyses, PiR-651 and piR-823 were both useful biomarkers for distinguishing GC from controls. A preferential biomarker with a higher positive likelihood ratio for CTC screening in GC was discovered to be PiR-823 (Cui et al., 2011; Sarkar et al., 2019). PiRNAs serve as highly sensitive biomarkers for gastric cancer screening, offering significant indicators to distinguish gastric cancer from control subjects. Peripheral blood biomarkers PiR-651 and piR-823 distinguish gastric cancer from controls, with expression decreasing in advanced stages, suggesting diagnostic potential, particularly in clinical stage I patients. PiR-5937 and piR-28876 expression dramatically dropped in the serum of colon cancer patients, according to the study, and had a high diagnostic potential for patients with clinical stage I. Nevertheless, following surgery, these levels rose, indicating a connection to the presence of the tumor. While piR-5937 and piR-28876 were downregulated in nearly 70% of samples, CEA/CA19-9 was found to be upregulated in less than 50% of patients (Vychytilova-Faltejskova et al., 2018).

PiRNAs such as piR-54265 may serve as valuable biomarkers for the early detection of colon cancer and post-surgical surveillance, demonstrating a positive correlation between tumor levels and the effectiveness of chemotherapy (Weng et al., 2019). Serum levels of a five-piRNA-based panel dropped from healthy controls to patients with colorectal adenoma, which may be a sign of early cancer progression. This panel may be useful in identifying people with familial adenomatous polyposis who are at a higher risk of colorectal cancer. The sensitivity, specificity, and AUC values of this panel were 0.782, 0.750, 0.862, 0.509, 0.9054, and 0.745, respectively, which demonstrated superior diagnostic potential compared to CEA-CA19-9-based Panel II (Qu et al., 2019). A stable marker in CRC patients, serum piR-54265 is upregulated in a stage-dependent manner and positively correlates with tumor levels; higher levels indicate a shorter survival period. Additionally, patients with lower levels respond better to chemotherapy, which is correlated with the treatment’s curative effectiveness.

The ROBERT study (Iliev et al., 2016a) revealed that piR-823 expression is altered in tumor tissue and serum levels in patients with renal cancer are increased and correlate with advanced clinical stages. PiRNA-823 demonstrates stability in peripheral blood extracellular vesicles, with heightened concentrations positively correlated with elevated levels of β2-microglobulin, serum creatinine, and hemoglobin, and negatively correlated with blood calcium and lactate dehydrogenase. PiR-651, a protein present in the serum of lymphoma patients, originates from circulating cells rather than neoplastic cells. In complete remission, there is no substantial difference in levels between patients and healthy controls (Iliev et al., 2016b; Li et al., 2019). PiRNA-823 is a long-term stability marker in peripheral blood EVs, especially in patients with renal injury and hyphemia or stage II and III lymphoma (MM). Elevated piRNA-823 is negatively correlated with blood calcium and LDH, but positively correlated with serum Cr, β2-MG, and Hb levels. PiR-651, which is downregulated in cHL patients’ serum, might be a reflection of variations in lymphoma-related peripheral blood populations. There is no discernible difference in levels between patients and healthy controls at full remission (Yan et al., 2015). The two primary stages of detection techniques for piRNAs as cancer biomarkers are usually targeted, quantitative validation and a high-throughput screening phase to find putative piRNAs. These techniques can be used to find a cancer signature in tissue and liquid biopsy samples, like blood serum or urine. Differentially expressed piRNAs between cancer patients and healthy controls are found using high-throughput techniques. The gold standard for initial discovery and expression profiling is small RNA sequencing (seq). Following the extraction of total RNA from tissue or liquid biopsy samples, libraries are created and Next-Generation Sequencing (NGS) platforms are used for sequencing. To detect and measure piRNA expression, the resultant sequence data is processed using bioinformatics tools and piRNA databases. Additionally, thousands of piRNAs can be profiled concurrently using microarrays to identify differences in expression between cancer samples and healthy controls (Taghizadeh et al., 2024).

PiRNAs, or small non-coding RNAs, can be found and measured in a number of ways. To verify the expression levels of individual piRNAs, the reverse transcription quantitative polymerase chain reaction (RT-qPCR) is a sensitive and precise technique. Using particular primers for the target piRNA, total RNA is amplified after being converted into complementary DNA (cDNA). To take into consideration variations in sample preparation, the expression levels are normalized to a stable internal control RNA. Custom TaqMan assays are RT-qPCR assays that are sensitive, specific, and commercially available. They are intended to measure individual piRNAs and other small non-coding RNAs. Droplet digital PCR (ddPCR) is a valuable technique for analyzing samples with low RNA content because it provides greater sensitivity and precision for quantifying piRNAs. An older, less accurate technique for confirming the existence and dimensions of particular RNA molecules, including piRNAs, is Northern blotting. Special protocols are needed for non-invasive liquid biopsies in order to extract and identify piRNAs from biofluids such as blood and urine. Extracellular vesicles (EVs), which are frequently stable in biofluids, are isolated during these processes. Commercial precipitation kits, size exclusion chromatography, and ultracentrifugation are common techniques. By detecting exosomal marker proteins like TSG101 and CD9, methods such as Western blot, transmission electron microscopy (TEM), and nanoparticle tracking analysis (NTA) are used to verify the quantity and quality of exosomes. New computational techniques like support vector machines and machine learning are improving the analysis of sizable piRNA datasets and forecasting piRNA-disease associations, which speeds up the search for biomarkers. The Catalytic Hybridization Assembly System (uniCHA) has proven to be highly effective in identifying biomarkers for breast cancer in plasma (Phay et al., 2018; Khavari et al., 2025; Ren et al., 2023; Xu et al., 2025).

### piRNAs as prognostic and therapeutic biomarkers in cancer

PiRNAs, comprising 132 identified in the gastric cancer genome, are associated with cancer prognosis. Almost fifty percent of these are overexpressed, with piRFR222326 linked to overall survival and five piRNAs significantly correlated with recurrence-free survival. HIWIL1 may serve as a valuable prognostic indicator for patients with hepatocellular carcinoma following curative resection. The expression of PIWIL4, PIWIL2, and PIWIL3 is associated with patient survival, tumor stage, and clinical parameters, indicating their potential as prognostic markers for tumors (Iliev et al., 2016b; AmeliMojarad and Amelimojarad, 2022; Kris et al., 2016).

Targeting PIWI proteins or modifying piRNA expression are two therapeutic approaches that use piRNA biomarkers in cancer to stop the spread, metastasis, or chemoresistance of the disease. Because piRNAs play a role in gene regulation and epigenetic modification, these strategies which are still in the early stages of research have promise for new diagnostic tools, prognostic markers, and targeted therapies in a variety of cancers (Mokarram et al., 2021). In order to potentially reverse cancer phenotypes like proliferation and invasiveness, therapeutic approaches for cancer include modifying piRNA expression, using synthetic molecules to target oncogenic piRNAs and inhibit their function, and restoring tumor-suppressive piRNAs to restore their expression. These tactics seek to identify and block piRNAs that promote cancer and suppress tumors. Potential strategies to stop the spread of cancer include antibody-based treatments and the transcriptional silencing of PIWI genes. While synthetic piRNAs can bind to PIWI genes, promoting their silencing and preventing the production of harmful proteins, antibodies can disrupt the interaction between PIWI proteins and piRNAs. Future treatments may involve modifying piRNA-mediated epigenetic mechanisms to reverse malignant phenotypes, as piRNAs can affect epigenetic factors that regulate gene expression and cancer progression (Cai et al., 2022). Examples of therapeutic applications include modifying piR-1245 for colon cancer, targeting piR-33733 for ovarian cancer, and investigating the role of piR-823 in HSP levels. Targeting PiR-017061, which interacts with PIWIL1, can inhibit the growth of cells in pancreatic cancer by promoting mRNA degradation. To create effective treatments, it is essential to comprehend the biogenesis and functions of piRNA. It is still difficult to create effective delivery methods for therapeutic piRNAs or antibodies. To confirm piRNAs as trustworthy biomarkers for early diagnosis, prognosis prediction, and treatment response tracking in a variety of cancers, more investigation is required (Yin et al., 2017; Xie et al., 2021).

## Concluding remarks and prospective directions

PiRNAs, identified in 2001, are integral to numerous species and physiological and pathological conditions. Nonetheless, altered expression may result in cancer progression. Specific trials have demonstrated that piRNAs exhibit abnormal expression patterns uniquely associated with various cancer types. Certain piRNAs are present in the blood or urine of cancer patients, serving as diagnostic or prognostic biomarkers. Multiple reports concentrate on tumor phenotype, resulting in unanswered questions. PiRNAs necessitate multi-center cohort studies with substantial sample sizes for clinical implementation in human oncology. The association between piRNAs and current therapies predominantly centers on chemotherapy, whereas their involvement in radiotherapy and immunotherapy is yet to be investigated. The advancement of multi-omics and high-throughput sequencing technologies is anticipated to facilitate a more thorough comprehension.

Advanced sequencing technologies facilitate the identification of various expressions of piRNAs/piwi proteins in cancer. Cancer represents a substantial global health challenge owing to its elevated morbidity and mortality rates. PiRNAs and Piwi proteins preserve a stable equilibrium in germ and somatic cells. The aberrant expression may result in cancer. PiRNAs, or Piwi-interacting RNAs, represent an expanding field of study, with databases such as piRBase and piRNA Bank offering resources for analyzing their functions and predicting target RNAs (Wang et al., 2019; Sai Laksh et al., 2008). Nevertheless, investigations into piRNAs have predominantly concentrated on transcriptional and post-transcriptional mechanisms, with limited research addressing post-translational modifications. The examination of post-transcriptional modifications of piRNAs is essential for elucidating tumorigenesis mechanisms, as they exhibit elevated expression in blood samples and demonstrate greater precision and sensitivity than traditional markers. PiRNAs are more sensitive and precise than conventional markers, and they may be used as therapeutic targets for CRISPR-Cas9-mediated genome editing, siRNA, and anti-sense oligonucleotides that suppress tumor growth.
